# Prescribing Trends of Glucagon-Like Peptide 1 Receptor Agonists for Type 2 Diabetes or Obesity

**DOI:** 10.1001/jamanetworkopen.2025.40890

**Published:** 2025-10-31

**Authors:** Piaopiao Li, Jithin S. Varghese, Megha K. Shah, Rodolfo J. Galindo, Francisco J. Pasquel, Mohammed K. Ali, Hui Shao

**Affiliations:** 1 Hubert Department of Global Health, Emory University Rollins School of Public Health, Atlanta, Georgia; 2 Department of Pharmaceutical Outcomes and Policy, University of Florida College of Pharmacy, Gainesville; 3 Emory Global Diabetes Research Center, Woodruff Health Sciences Center, Emory University, Atlanta, Georgia; 4 Department of Family and Preventive Medicine, School of Medicine, Emory University, Atlanta, Georgia; 5 Division of Endocrinology, Miller School of Medicine, University of Miami, Miami, Florida; 6 Division of Endocrinology, Emory University School of Medicine, Atlanta, Georgia

## Abstract

This cross-sectional study examines the US prescribing trends of glucagon-like peptide 1 (GLP-1) receptor agonists among individuals with type 2 diabetes and/or obesity.

## Introduction

Research has highlighted the benefits of glucagon-like peptide 1 receptor agonists (GLP-1RAs) in managing blood glucose and reducing weight in individuals with type 2 diabetes (T2D) and/or obesity.^1^ However, current trends on how these drugs are prescribed across indications and subpopulations remain unknown. This study examined prescribing trends of GLP-1RAs among individuals with T2D and/or obesity.

## Methods

This cross-sectional study used an integrated electronic health record (Epic Cosmos; Epic Systems Corp) covering more than 300 million US residents.^2^ The Emory University Institutional Review Board exempted this study from review and informed consent as this was a secondary analysis of deidentified data. The study followed the STROBE reporting guideline.

**Figure 1.**
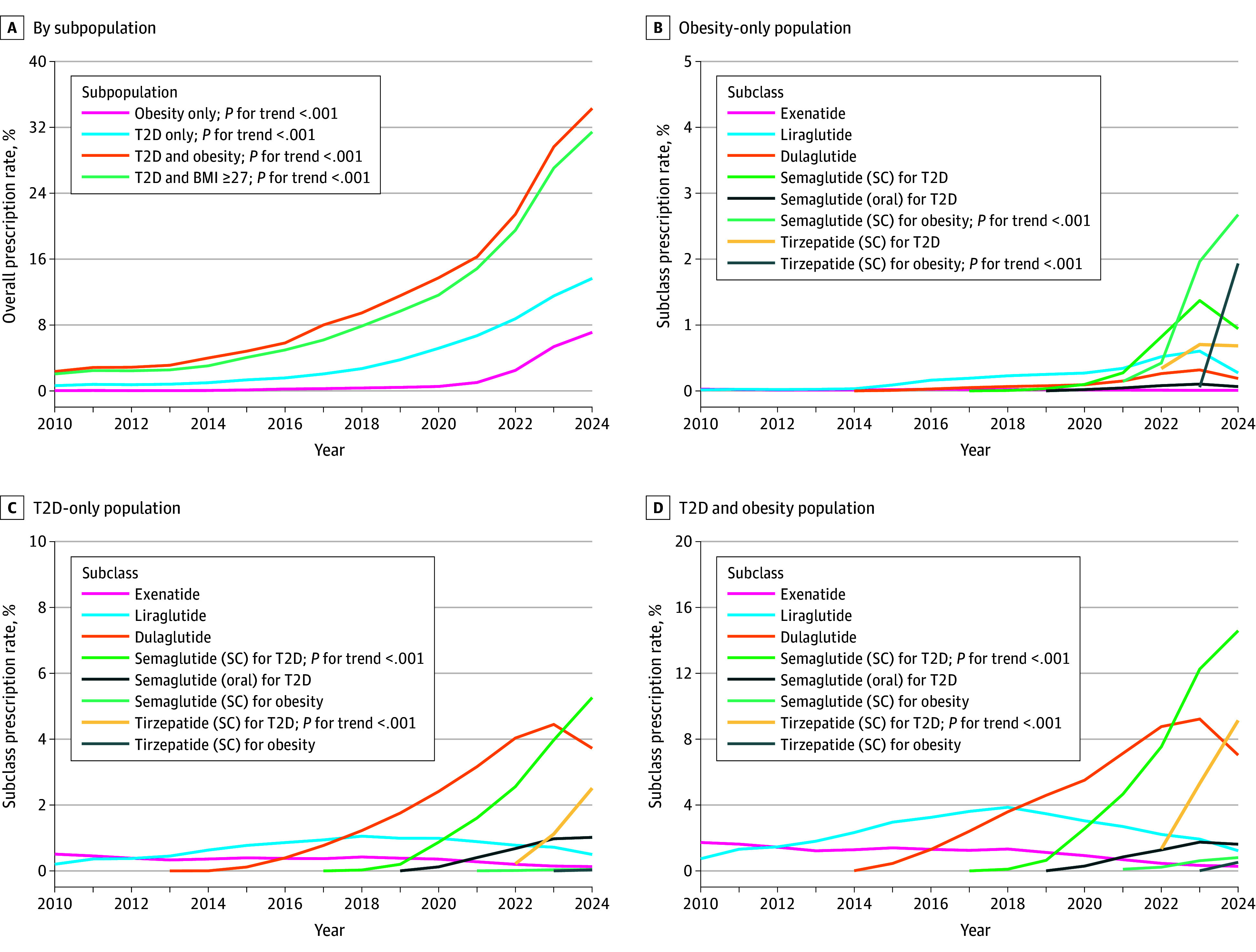
Annual Glucagon-Like Peptide 1 Receptor Agonist (GLP-1RA) or Dual Glucose-Dependent Insulinotropic Polypeptide and GLP-1RA Prescription Rates Among Different Subpopulations, 2010 to 2024 Note that dulaglutide showed a comparable rate to semaglutide, possibly reflecting longer market availability or formulary preference. *P* for trend values are reported only for groups showing the most notable increases. BMI indicates body mass index (calculated as weight in kilograms divided by height in meters squared); SC, subcutaneous; T2D, type 2 diabetes.

We examined trends in GLP-1RA prescriptions from January 1, 2010, to January 1, 2025, among subpopulations with T2D only, obesity only, and T2D and obesity. Prevalent T2D was identified using DiCAYA algorithms.^3^ Obesity was determined by diagnosis code or body mass index (BMI) (weight in kilograms divided by height in meters squared) of 30 or higher. We included a subgroup with T2D and BMI of 27 or higher. The outcome was the proportion of individuals prescribed a GLP-1RA (Figure 1) each year.

 We calculated the Gini index to measure differences in prescription rates in 2024 by sex, age, self-reported race and ethnicity, social vulnerability index, rural-urban commuting area, and insurance type. Trend analysis was conducted by fitting linear regressions (eMethods in Supplement 1). Statistical analyses were performed using R, version 4.2.3 (R Foundation). A 2-sided *P* < .05 was considered significant.

## Results

We identified 17.9 million individuals with T2D only (mean [SD] age, 68.6 [13.9] years; 46.6% female and 53.4% male), 59.8 million with obesity only (mean [SD] age, 48.7 [18.9] years; 60.4% female and 39.6% male), and 12.9 million with T2D and obesity (mean [SD] age, 62.9 [13.9] years; 53.0% female and 47.0% male). From 2010 to 2024, prescription rates increased substantially, with the most growth in the T2D and obesity group (2.4% to 34.3%), followed by the T2D and BMI of 27 or higher (2.1% to 31.5%), T2D-only (0.6% to 13.7%), and obesity-only (0.04% to 7.1%) groups (Figure 1). In the obesity-only group, semaglutide prescriptions increased from 0.4% in 2022 to 2.7% in 2024, followed by tirzepatide from 0.06% in 2023 to 1.9% in 2024. In the T2D-only group, semaglutide prescriptions grew from 0.2% in 2019 to 5.3% in 2024, and tirzepatide from 0.2% in 2022 to 2.5% in 2024. A similar trend was observed in the T2D and obesity group, with semaglutide prescriptions (subcutaneous, for T2D) increasing from 0.6% in 2019 to 14.6% in 2024 and tirzepatide (for T2D) from 1.3% in 2022 to 8.1% in 2024.

**Figure 2.**
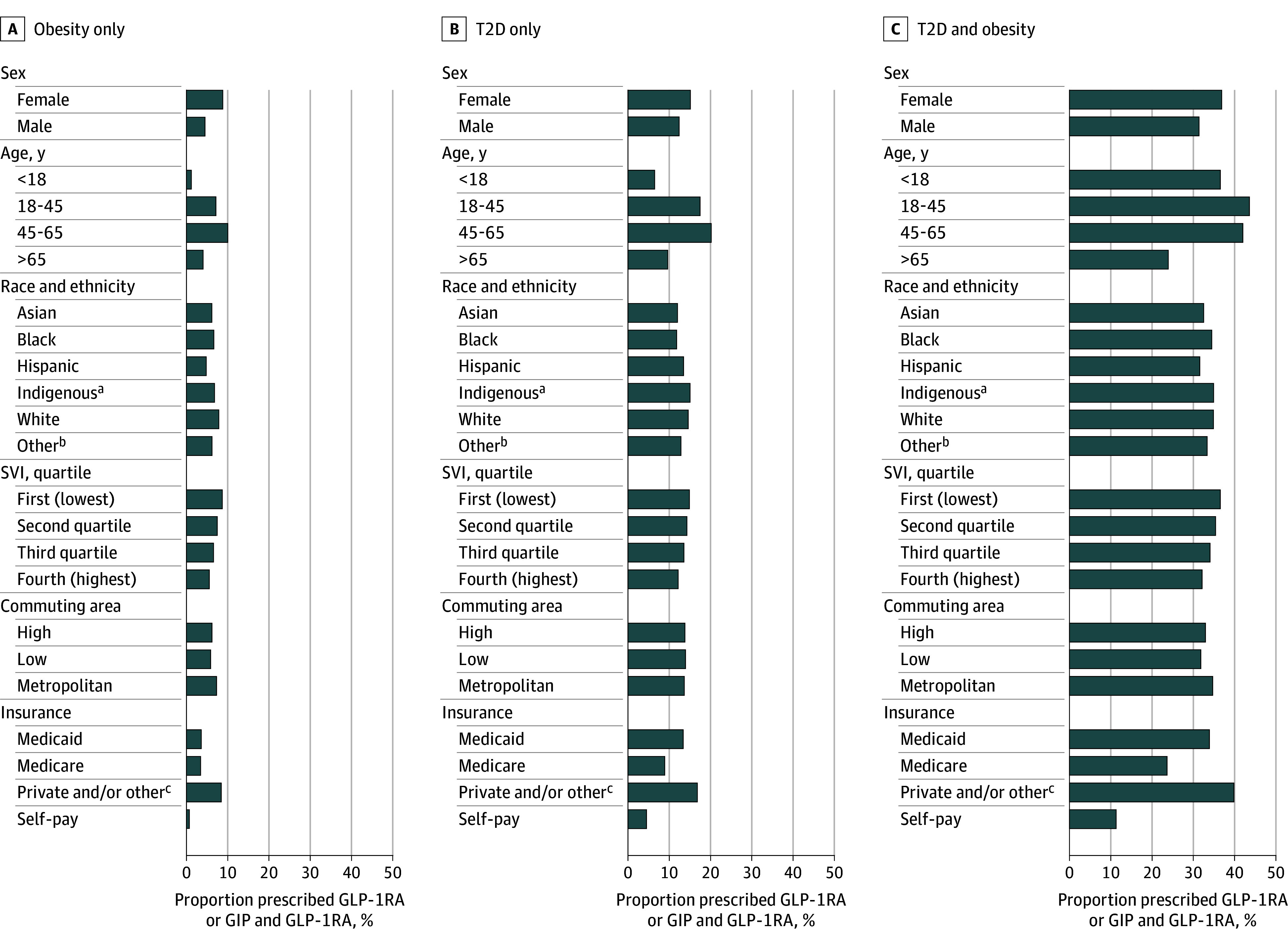
Differences in Glucagon-Like Peptide 1 Receptor Agonist (GLP-1RA) and Dual Glucose-Dependent Insulinotropic Polypeptide (GIP) and GLP-1RA Prescriptions Among Subpopulations in 2024 Differences were measured using the Gini index. The social vulnerability index (SVI) is a tool developed by the Centers for Disease Control and Prevention to help public health officials identify communities that are more vulnerable to external stresses on human health. A high SVI score indicates greater vulnerability. Race and ethnicity were self-reported. T2D indicates type 2 diabetes. ^a^American Indian or Alaska Native and Native Hawaiian or Pacific Islander. ^b^Other includes multiracial or multiethnic or those with missing information. ^c^Other health insurance includes unspecified or unknown, Medicare Advantage, Medicare Replacement, Tricare, Worker’s Compensation, Veteran’s Affairs, and third-party liability.

In 2024, insurance-based differences in prescriptions were observed (Gini indices: obesity only, 0.48; T2D only, 0.32; T2D and obesity, 0.29) (Figure 2). Sex-based differences were most notable in the obesity-only group (Gini index, 0.32). Age-based differences were present across groups (Gini index: obesity only, 0.44; T2D only, 0.30; T2D and obesity, 0.15).

## Discussion

This cross-sectional study found an increase in GLP-1RA prescriptions, with notable differences across subpopulations by insurance type, sex, and age. Tirzepatide and semaglutide grew the fastest, possibly due to their superior glycemic, weight loss, and guideline-emphasized cardiorenal benefits.^4^ Although GLP-1RAs are generally covered for T2D, coverage for obesity is limited (eg, Medicare excludes antiobesity drugs). Off-label semaglutide (for T2D) use in the obesity-only group underscores access barriers.^5^ Higher prescription rates among privately insured individuals indicate that socioeconomic factors influence access.^6^ Despite evidence about weight management, prescriptions remain low among people with obesity only. Study limitations included that medications were recorded based on prescriptions, not dispensations; variation in health system data contribution duration to Cosmos may have biased the trend analysis; and missing BMI may have led to underidentification of obesity, potentially limiting generalizability. This study highlights increasing GLP-1RA prescriptions and sociodemographic disparities, underscoring the need for policies to improve access, particularly among people with obesity only.

## Notes

Supplement 1.
eMethods.

eReferences.

Supplement 2.
Data Sharing Statement
