# Supplementary material for: Prescribing Trends of Glucagon-Like Peptide 1 Receptor Agonists for Type 2 Diabetes or Obesity
Source: JAMA Netw Open. 2025 Oct 31;8(10):e2540890. doi: 10.1001/jamanetworkopen.2025.40890 (PMC12579341; doi:10.1001/jamanetworkopen.2025.40890)
Supplement: Supplement 1. — eMethods. eReferences. [file jamanetwopen-e2540890-s001.pdf]

Supplemental Online Content

Li P, Varghese JS, Shah MK, et al. Prescribing trends of glucagon-like peptide 1 receptor agonists for type 2 diabetes or obesity. *JAMA Netw Open*. 2025;8(10):2540890. doi:10.1001/jamanetworkopen.2025.40890

eMethods

eReferences

This supplemental material has been provided by the authors to give readers additional information about their work.

© 2025 Li P et al. *JAMA Network Open*.

eMethods

Database: Cosmos is a data platform that integrates with the Epic electronic health record (EHR) system. Organizations that use Epic software to provide direct patient care are eligible to join the Cosmos community. Cosmos has been accepting data since 2018, with historical (backloaded) data extending as far back as 2005. The coverage of the population has increased continuously. As of Jun 2024, Cosmos has data from more than 257 million unique patients, 12.2 billion encounters with representation from all 50 states. The contributions come from about 250 participating sites, which include more than 1,500 hospitals, more than 34,000 clinics and 289,000 hospital beds, more than 363,000 physicians. Cosmos data aligns closely to population metrics of the US census and has a representative sample of patients across race, ethnicity, sex, age, types of insurance, and social vulnerability index.[1] All data are specific to the Cosmos database and are not extrapolated to represent national totals.

DiCAYA algorithm: In 2020, CDC and the National Institutes of Diabetes and Digestive and Kidney Diseases jointly funded the Diabetes in Children, Adolescents and Young Adults (DiCAYA) Network through 2025. The DiCAYA Network aims to advance the efficiency, flexibility, sustainability and transportability of diabetes surveillance efforts through the use of large-volume EHR data.[2] An EHR-based computable phenotype algorithm was developed by the team for accurate identification of incident and prevalent T1D and T2D.[3] Briefly, All individuals with any indication in the EHR of possible diabetes are identified by applying the following criteria: (1) ⩾1 haemoglobin A1c⩾6.5%; (2) ⩾1 fasting glucose ⩾126 mg/dL; (3) ⩾1 random plasma glucose⩾200 mg/dL; (4) ⩾1 diabetes-related diagnosis code from an inpatient or outpatient encounter or (5) ⩾1 prescribed, administered or dispensed medication that is typically indicated for the treatment of diabetes. The computable phenotype is employed on those with at least one diabetes diagnosis code. Diabetes type (type 1, type 2, other) will be defined based on the proportion of diabetes type-specific diagnosis codes (type 1, type 2 or other) among total diabetes codes, using plurality to assign type. In ties, type 1 is given preference over type 2, and type 2 is given preference over others. The sensitivity, specificity, PPV and NPV is 94%, 95%,, and 96%

© 2025 Li P et al. *JAMA Network Open*.

respectively for those who are aged < =18 years. The sensitivity, specificity, PPV and NPV is 93%, 84%, and 95% respectively for those who are aged > 18 years.

This was a pooled cross-sectional analysis using electronic health record data from the Epic Cosmos database. We examined the trends of GLP-1RA and GIP/GLP-1RA (albiglutide, dulaglutide, exenatide, liraglutide, lixisenatide, semaglutide and tirzepatide) prescription in three population subgroups identified in Cosmos from 2010 to 2024: T2D-only, obesity-only, and T2D with obesity. We examined the prescription by subclass of GLP-1RA and GIP/GLP-1RA (exenatide, liraglutide, dulaglutide, semaglutide and tirzepatide) within each subpopulation. We further explored the prescription of GLP-1RA and GIP/GLP-1RA by sociodemographic characteristics including sex, age, race/ethnicity, social vulnerability index, rural-urban commuting area, and insurance coverage, within each subpopulation. The disparities in prescription were measured using the Gini index. The outcome was the percentage of the subpopulation who were prescribed GLP-1RA and GIP/GLP-1RA in each calendar year. Individuals with T2D at any age were identified using the DiCAYA algorithm. Obesity status was determined based on International Classification of Diseases diagnosis codes or body mass index measurements (BMI>= 30 kg/m^2). This approach ensured that individuals were not misclassified solely due to missing BMI. Medication prescription was identified by RxNorm codes. To be included in the analysis and counted toward the denominator for a given year, individuals with T2D or obesity must have at least one electronic health record encounter in that year.

Race and ethnicity were reported by the patients. Race and ethnicity were categorized as Asian, non-Hispanic Black, Hispanic, Non-Hispanic White, Native, and Other. Native group includes Native Hawaiian, Pacific Islander, American Indian, and Alaska Native. Age was categorized as <18 years, 18-45 years, 45-65 years, and >65 years. Social Vulnerability Index (SVI) is a tool developed by the US Centers for Disease Control and Prevention to help public health officials identify communities that are more vulnerable to external stresses on human health. SVI was grouped based on quartile. The area was grouped as a high commuting area, low commuting area, and metropolitan based on rural-urban

© 2025 Li P et al. *JAMA Network Open*.

commuting area codes. We conducted trend analysis to assess the temporal changes of prescription rates by fitting a linear regression model of prescription rates over the calendar years. The p-values for the trend were calculated by testing the null hypothesis that the annual change in prescription rates was zero. 2-sided $P < .05$ was considered statistically significant. In sensitivity analyses for the prescription trend, we included polynomial terms (quadratic and cubic terms) for calendar year to account for nonlinearity in prescription trajectories. We utilized Microsoft SQL Server Management Studio (v18.5.1) to extract data from Cosmos, and conducted subsequent analyses using R version 4.2.3.

eReferences

1. Epic Systems Corporation. https://cosmos.epic.com/.

2. Hirsch AG, Conderino S, Crume TL, et al. Using electronic health records to enhance surveillance of diabetes in children, adolescents and young adults: a study protocol for the DiCAYA Network. *BMJ Open*. 2024;14(1):e073791. doi:10.1136/bmjopen-2023-073791

3. Shao H, Thorpe LE, Islam S, et al. Developing a Computable Phenotype for Identifying Children, Adolescents, and Young Adults With Diabetes Using Electronic Health Records in the DiCAYA Network. *Diabetes Care*. Published online March 31, 2025:dc241972. doi:10.2337/dc24-1972

© 2025 Li P et al. *JAMA Network Open*.
