# Supplementary material for: Prescribing Trends of Glucagon-Like Peptide 1 Receptor Agonists for Type 2 Diabetes or Obesity
Source: JAMA Netw Open. 2025 Oct 31;8(10):e2540890. doi: 10.1001/jamanetworkopen.2025.40890 (PMC12579341; doi:10.1001/jamanetworkopen.2025.40890)
Supplement: Supplement 2. — Data Sharing Statement [file jamanetwopen-e2540890-s002.pdf]

Data Sharing Statement

Data
Data available: No

Additional Information
Explanation for why data not available: The data cannot be shared by authors. Cosmos is built by a community of health systems using Epic software and agreeing to collaborate. Direct access to Cosmos is available for those affiliated with and approved by a Cosmos participating organization.
